# Giant cell tumor: Curettage and bone grafting

**DOI:** 10.4103/0019-5413.32042

**Published:** 2007

**Authors:** Dominic Puthoor, Wilson Iype

**Affiliations:** Dept. of Orthopedics, Amala Institute of Medical Sciences, Amalanagar, Thrissur, Kerala - 680 555, India

**Keywords:** Computerized tomography scan, curettage and bone grafting, wide resection

## Abstract

**Background:**

Curettage and wide resection are accepted methods of treatment of giant cell tumor (GCT) of bone. The success rate with curettage in different reports varies widely. There is a paucity in the literature regarding selection of cases for curettage. Present study is an analysis of outcome of 34 cases treated by curettage and bone grafting.

**Materials and Methods:**

Thirty-four cases of GCT of bone, 28 fresh and six with recurrence were treated by curettage and bone grafting. All cases of Campanancci grade 1, 2 and grade 3 which on computerized tomography scan showed break in the cortex confined to one surface and cortical break less than one third of circumference were treated by curettage and bone grafting.

**Results:**

4 (14%) of these lesions treated primarily by us showed recurrence after one and half year.

**Conclusion:**

Curettage and bone grafting is a reliable method in the treatment of GCT, provided guidelines regarding selection of cases and principles of tumor surgery are strictly adhered to.

Giant cell tumor (GCT) of bone is a benign but locally aggressive tumor that usually involves the ends of long bones. It occurs most frequently in the third decade of life, i.e. after physeal plate closure. The lesion consists of multinucleated giant cells mixed with mononuclear stromal cells. They represent 20% of all benign bone tumors and 5% of all bone tumors.^1^ High incidence is seen in China and India, where they represent up to 20% of all bone tumors.^2^,^3^

GCT of the bone has an unpredictable behavior, not always related to radiographic or histological appearance.^4^ This makes the treatment of the disease a subject of constant debate. The best treatment should ensure local control of disease and maintain function. Curettage has been the preferred treatment for most cases of GCT. Many earlier studies had shown very high (25-50%) local recurrence rates after curettage and bone grafting.^3^–^5^ The use of modern imaging techniques and extended curettage through the use of power burrs and local adjuvants have improved outcome with reduced recurrence rates (10-20%). Phenol, liquid nitrogen, bone cement, hydrogen peroxide, zinc chloride and more recently, argon beam cauterization have been employed as local adjuvants. Chemical or physical agents work by inducing an additional circumferential area of necrosis to “extend” the curettage.^1^

We present the outcome of GCT of the bone treated by curettage and bone grafting since 1989 to highlight the usefulness of CT scan in selecting cases for curettage and grafting and the choice of the surgical approach.

## MATERIALS AND METHODS

Fifty-two patients treated by us during 1989-2004 constituted the clinical material. Thirty-two of our cases were around the knee joint. Most of our patients (35 cases) were in the third decade. There were 21 males and 31 females. Apart from routine investigations such as Hb%, TLC, DLC, ESR, S. Calcium, S. Alkaline phosphatase, X-ray of the lesion and X-ray chest, all patients were subjected to CT scan. Diagnosis was established by CT-guided core biopsy.

**Figure 1:**
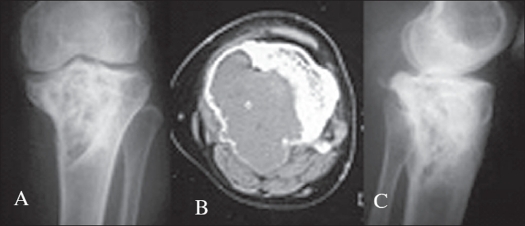
Campanacci Stage 3. This case was treated by curettage and bone grafting in 1997. A) CT scan before surgery. B) and C) X-ray taken in 2003. Review in 2005 shows no evidence of recurrence

Cases were classified according to Campanacci's grading system.^2^,^4^ Procedure to be selected was decided based on CT scan findings. All cases which on CT scan showed break in the cortex confined to one surface [Figure 1] and cortical break less than one-third of its circumference, were treated by curettage and grafting. All cases belonging to Campanacci's Grade 1 and 2 as well as cases belonging to Grade 3 which fulfilled the above criteria were treated by curettage and grafting. Twenty-eight primary lesions and six cases of recurrences were treated by curettage. Bone graft was used to fill up the resultant cavity in all except four cases where bone cement was used. Present analysis is about these 34 cases underwent curettage and bone grafting.

The lesions in these 34 cases affected, upper tibia (n=15), lower femur (n=10), upper humerus (n=3), lower radius (n=3), calcaneum, talus and first metacarpal in one each.

For curettage, the lesion was approached through a site of cortical break. In a lesion of lower femur and upper tibia, if the break in the cortex was in the posterior aspect [Figure 1], posterior approach, isolating popliteal vessels and tibial nerve was preferred. In our series we went through posterior approach in four cases of lower femur and six cases of upper tibia. In the rest of the cases of lower femur and upper tibia, the approach was anteromedial or anterolateral depending upon the cortical break in the CT scan. In a case of GCT calcaneum, the cortical break was on its superior nonarticular surface. We detached the insertion of tendo calcaneus for proper curettage and later repaired by using pullout sutures. Thus a wider area of tumor removal at the site of cortical break was achieved, where there was tumor extension to extraosseous tissues. All six cases of recurrence were initially treated elsewhere. Out of these four cases underwent treatment initially with out CT scan and in remaining two cases though CT scan was taken, approach was not through the area of cortical defect.

After exposure, the site of the cortical break was identified by palpation and a circumferential area of 1 cm × 1 cm beyond the margin of the cortical break is marked using a cautery. With a small osteotome, the cortex is broken and with scissors, the area where there is soft tissue extension is removed as a lid, taking care not to spill the tumor. The cavity after thorough curettage, is washed several times with hydrogen peroxide and saline. The cavity was cauterized with phenol and then tightly filled with bone graft. The cases were followed up at six-week intervals until six months and then at three-month intervals till one year and then at six-month intervals.

## RESULTS

**Figure 2:**
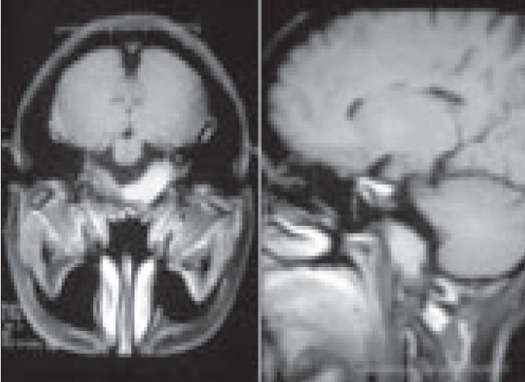
Secondary deposit from GCT lower radius in the clivus

Maximum follow-up was 17 years and minimum two years with a mean follow-up of six years. In patients treated by curettage and grafting, functional evaluation was done after four months according to Enneking's method that takes into consideration range of movement of the joint, pain, stability, deformity, muscle strength, functional activity and subjective opinion. It was found that all cases showed good function.^6^ Of the 28 primary cases treated by curettage and bone grafting, four had recurrence. Of these none of them recurred before one and a half years. One case of recurrence occurred after six years. Two cases belonged to Stage 2 of Campanacci's grading system and two cases belonged to Stage 3. One such recurrent case in the lower femur was treated by a custom prosthesis. One case of upper tibia underwent resection arthrodesis. Another case of upper tibia did not come to us for further treatment. Fourth one was in lower radius; it was treated by Enbloc resection and reconstruction with non vascularized proximal fibula.^7^,^8^ Out of six recurrent cases (all had their first surgery in other hospitals) treated by us by second curettage, four recurred. Both the first and second recurrence occurred before one year which is much earlier than the cases of recurrence we had in our primary cases. Two cases of upper end tibia out of the four opted for above knee amputation. One case of lower end femur opted for radiation therapy and one was treated by custom prosthesis. In three cases, recurrences occurred during pregnancy. This happened after one and a half years, three years and four years respectively. No case in the curettage group had lung metastasis.^9^ A 21-year-old lady, who presented with a second recurrence in the lower radius, was treated by wide excision. She presented to us with deviation of tongue to one side, one year after the third surgery. Investigation showed secondary deposit in the base of skull (clivus) producing compression of hypoglossal nerve [Figure 2]. Biopsy confirmed the diagnosis of secondary deposit from GCT.

## DISCUSSION

Curettage and wide resection have been the accepted methods of treatment for GCT of bone.^1^–^4^ Turcotte considers that many Stage 3 tumors, multiple local recurrences and pathologic fracture when joint anatomy cannot be restored are better treated with wide resections.^1^ The definite criteria that guide the orthopedic surgeon to decide whether a particular case is to be treated by curettage have not been defined adequately. In this study we are suggesting some guidelines based on the CT finding for helping the surgeon to decide whether a particular case is to be treated by curettage or does it require resection.

The CT scan has significant role in the management of GCT as it helps in a) CT-guided core biopsy; b) Knowing the site of cortical break and the resultant soft tissue extension; c) Deciding the surgical approach. We performed curettage in cases where the break in cortex was confined to only one surface and the break did not exceed one-third circumference of bone.

We approached the tumor through an area of cortical break so as to achieve complete tumor clearance. The higher incidence of recurrence after curettage and bone grafting reported earlier is partly also because of the defective approach. If a tumor around the knee joint even if the break in the cortex is posteromedial, the lesion is approached from the lateral side. This inevitably leads to failure to clear the area of soft tissue extension on postero medial side. Moreover, there is a contamination of the soft tissue during surgery on the lateral side. This may be the reason for a higher rate of second recurrence in the cases which presented to us with first recurrence. Recurrence rate in the 28 cases which had curettage as the primary procedure was 14%, almost similar to the recurrence rate which is reported in the literature.

## CONCLUSION

In GCT of bone, curettage and bone grafting still remains the ideal treatment (Campanacci Gr I, Gr II and selected cases of Gr III). Low rate of recurrence can be achieved if curettage and grafting is done in properly selected cases through a well-planned surgical approach after assessing the cross-sectional CT.
